# Surgical management of pancreatic neoplasms in children: a single-institution experience over 15 years

**DOI:** 10.3389/fped.2024.1468276

**Published:** 2024-09-17

**Authors:** In Geol Ho, Kyong Ihn, Sung Min Le, Soyong Shin, Seok Joo Han

**Affiliations:** Division of Pediatric Surgery, Severance Children’s Hospital, Department of Surgery, Yonsei University College of Medicine, Seoul, Republic of Korea

**Keywords:** pediatric pancreatic neoplasm, pancreatic tumor, surgical resection, pancreatectomy, surgical outcome

## Abstract

**Introduction:**

Pancreatic neoplasms are rare among children and very few studies have reported on surgical outcomes for pediatric pancreatic neoplasms. Therefore, we aimed to describe patient and tumor characteristics and report on the surgical outcomes of pediatric pancreatic neoplasm.

**Methods:**

In this retrospective single-center study, we reviewed and analyzed the data of patients who underwent surgery for pediatric pancreatic neoplasms at Severance Children's Hospital between January 2007 and December 2022. Clinical data including demographics, surgical procedures, and postoperative and long-term outcomes were evaluated.

**Results:**

A total of 28 patients underwent surgical treatment for pancreatic neoplasms with a median age of 11.7 years (range: 0.4–17.8). The most common histological diagnosis among benign tumors was solid pseudopapillary neoplasm (SPN), which occurred in 20 patients (71.4%). This was followed by a mucinous cyst, nesidioblastosis, pseudocyst, duplication cyst, and benign cyst, each occurring in one patient (3.5%). Regarding malignant tumors, pancreatoblastoma, solid pseudopapillary carcinoma, and malignant pheochromocytoma were noted in one patient each (3.5%). Tumor locations included the head in 4 patients (14.2%), the body in 7 (25%), and the tail in 16 (57.1%), and was diffuse in 1 (3.5%). The most common surgical resection range was distal pancreatectomy, found in 22 patients (78.5%), followed by pylorus-preserving pancreaticoduodenectomy, found in 2 (7.2%); duodenum-preserving pancreatic resection, central pancreatectomy, tumor enucleation, and near-total pancreatectomy were performed in one patient each (3.5%). Overall, 4 patients developed grade B or C postoperative pancreatic fistulas, and 1 experienced postoperative mortality due to uncontrollable bleeding. The mean follow-up period was 6.1 years (range: 1–15.6 years), during which no significant impact on growth after surgery was detected. Among the 20 patients with SPN, tumor rupture occurred in 4 (20%), among whom 2 experienced tumor recurrences.

**Conclusions:**

Histological diagnosis of benign tumors was predominant in this case series and various extents of surgical resection were performed. Surgical treatment for pediatric pancreatic neoplasms appears to be safe and effective. However, considering the long-term prognosis of these patients, it is essential to determine the appropriate extent of surgical resection based on the location of the tumor.

## 1 Introduction

Pancreatic neoplasms in children are very rare, representing 0.2% of all pediatric malignancies (1). Unlike the adult presentation of pancreatic cancer, the common histologic diagnoses in pediatric patients have a different spectrum and more favorable outcomes (2, 3). Additionally, pancreatic adenocarcinomas are extremely infrequent in the pediatric population than in the adult population (4). Pancreatoblastomas are more frequent during the first decade of life, whereas solid pseudopapillary neoplasms (SPNs) are common in adolescence (5). Other less common neoplasms such as neuroblastoma, neuroendocrine tumors, rhabdomyosarcoma, and lymphoma have also been reported (1, 6, 7). In children, pancreatic neoplasms are generally asymptomatic and discovered incidentally during medical examinations for other conditions. Patients often present with non-specific symptoms such as abdominal pain, vomiting, palpable mass, or weight loss (8) and the mainstay for pancreatic neoplasms is surgical treatment regardless of histological diagnosis (except in lymphoma) (9). In adults, the overall morbidity and mortality rates associated with pancreatectomies have been extensively studied; however, limited information is available on the outcomes of pancreatectomies in pediatric patients. Therefore, this study aimed to review the management of pancreatic neoplasms in children with an emphasis on their presentation, diagnosis, treatment, and outcomes.

## 2 Materials and methods

In this retrospective single-center study, we analyzed the clinical data of patients aged 0 to 18 years who had undergone surgical resection for the preoperative diagnosis of pancreatic neoplasms at Severance Children's Hospital between January 2007 and December 2022. All patients included in our study were treated by pediatric surgeons. Information on patient demographics, clinical symptom presentation, tumor location, tumor size, pathologic results, and extent of resection were collected. Intraoperative and perioperative morbidity and mortality as well as postoperative height and weight development were analyzed. This study was approved by the Institutional Review Board of Yonsei University College of Medicine (approval number: 4-2023-0665). The requirement for informed consent was waived because of the retrospective nature of the study.

### 2.1 Statistical analysis

All data were analyzed using SPSS version 25.0 (IBM Corp., Armonk, NY, USA). Continuous data are presented as means and standard deviations (SD). Categorical data were compared using a one-way analysis of variance (ANOVA) or the *t*-test for normally distributed data and the Mann–Whitney *U* test for non-normally distributed data. A *p*-value of less than 0.05 was considered statistically significant.

## 3 Results

### 3.1 Demographics

**Figure 1:**
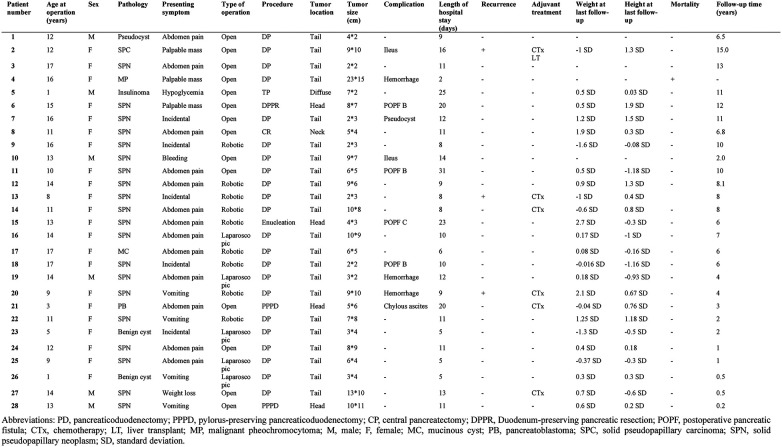
Patient characteristics.

The demographic characteristics of pediatric patients who underwent surgical treatment for pancreatic neoplasms are summarized in Table 1; Figure 1. A total of 28 pediatric patients underwent treatment for pancreatic neoplasms at Severance Children's Hospital during the study period. Among them, 21 were female (75%) and 7 were male (25%); the median age was 11.7 years (range: 0.41–17.8 years).

**Table 1:** Demographics of patient characteristics.

Characteristic	Number (*N* = 28)
Sex (F:M)	21:7
Median age at surgery (years)	11.7 (0.41–17.8)
Median tumor size (cm, range)	6.8 (2–23)
Presenting symptom	
Abdominal pain	13 (46.4%)
Incidental finding	5 (17.8%)
Palpable mass	3 (10.7%)
Vomiting	4 (14.2%)
Weight loss	1 (3.5%)
Bleeding	1 (3.5%)
Hypoglycemia	1 (3.5%)
Location of tumor in the pancreas	
Head	4 (14.2%)
Body	7 (25%)
Tail	16 (57.1%)
Diffuse	1 (3.5%)
Neoadjuvant chemotherapy	1 (3.5%)
Adjuvant chemotherapy	6 (21.4%)
Median follow-up period (years)	6.1 (1–15.6)

### 3.2 Preoperative evaluation

The main presenting symptom among patients was abdominal pain (*n* = 13, 46.4%), followed by incidental finding (17.8%), palpable mass (10.7%), vomiting (14.2%), and weight loss, bleeding, and hypoglycemia (3.5%). Tumor locations were in the head, body, and tail in 4 (14.2%), 7 (25%), and 16 (57.1%) patients, respectively, and the tumor was diffuse in 1 (3.5%) patient. Neoadjuvant chemotherapy was administered to 1 patient with pancreatoblastoma (3.5%) and adjuvant chemotherapy was administered to 6 (21.4%). The mean follow-up period was 6.1 years (range: 1–15.6 years) (Table 1).

### 3.3 Surgical procedure

Surgical procedures and outcomes were evaluated based on tumor location (head, body, tail, or diffuse) (Table 2). Each patient was investigated preoperatively using computed tomography and/or magnetic resonance imaging scans. The mean tumor size was 6.8 ± 4.4 cm. The surgery was performed with the principle of minimal resection of pancreas, depending on the location and size of the tumor. Based on the anatomical landmark of the SMV (superior mesenteric vein (SMV), procedures including pylorus-preserving pancreaticoduodenectomy (PPPD), duodenum-preserving pancreatic resection (DPPR), central pancreatectomy (CP), or distal pancreatectomy (DP). were selectively performed. The median tumor size was 6.75 cm (range: 4–10 cm), 6.29 cm (range: 2–13 cm), and 6.81 cm (range: 2–33 cm) in the head, body, and tail, (diffuse cannot be measured) respectively, with no significant difference between each location (*p* = 0.995). Open, robot-assisted, and laparoscopic surgeries were performed in 14 (50%), 9 (32.1%), and 5 (17.8%) patients, respectively.

**Table 2:** Surgical treatments and outcomes.

	Location of tumor (*N*)				
	Head (4)	Body (7)	Tail (16)	Diffuse (1)	*p*-value^a^
Median tumor size (range, cm)	6.75 (4–10)	6.29 (2–13)	6.81 (2–23)	Not measurable	0.995
Surgical method					0.538
Open	3	2	8	1	
Robotic	1	4	4	0	
Laparoscopic	0	1	4	0	
Operation time (min)	495 ± 90	497 ± 118	355 ± 161	420	0.257
Blood loss (ml)	592 ± 250	438 ± 258	414 ± 233	70	1.36
Range of surgical resection					0.08
NT	0	0	0	1	
DP	0	6	16	0	
PPPD	2	0	0	0	
DPPR	1	0	0	0	
CP	0	1	0	0	
Enucleation	1	0	0	0	
Complication					0.254
Wound complication	1	0	2	0	
Ileus	0	0	2	0	
POPF (grade B,C)	4	1	1	0	
Hemorrhage	0	2	1	0	
Chylous ascites	1	0	0	0	
Pseudocyst	0	0	0	0	
Clavien–Dindo classification (Grade)					0.368
I	0	0	0	0	
II	3	3	6	0	
III	1	0	0	0	
IV	0	0	0	0	
V	0	0	1	0	
Mean length of hospital stay (days)	18.5 ± 5.1	9.86 ± 22	10.8 ± 6.5		0.485
Tumor rupture					0.326
Before surgery	0	0	1		
During surgery	0	2	1		
Recurrence	0	1	2		0.254
Adjuvant CTx	1	3	2		0.471
Mortality	0	0	1		0.478
Median weight at last follow-up (Z-score, range)	0.94(−0.4–2.7)	0.38(−1.6–2.1)	0.09(−1.3–2.7)		0.487
Median height at last follow-up (Z-score, range)	0.6(−0.3–1.9)	−0.1(−1.1–0.8)	0.04(−1.1–1.3)		0.915

NT, near-total pancreatectomy; DP, distal pancreatectomy; PPPD, pylorus-preserving pancreaticoduodenectomy; DPPR, duodenum-preserving pancreatic resection; CP, central pancreatectomy; POPF, postoperative pancreatic fistula; CTx, chemotherapy.

^a^ Data were analyzed using a one-way analysis of variance (ANOVA).

The operation time was 495 ± 90 min, 497 ± 118 min, 355 ± 161 min, and 420 min and blood loss was 592 ± 250 ml, 438 ± 258 ml, 414 ± 233 ml, and 70 ml in the head, body, tail, and diffuse area, respectively, with no significant difference between each location (*p* = 0.257 and *p* = 1.36). The extent of surgical resection was distal pancreatectomy in 22 (78.5%) patients, followed by pylorus-preserving pancreaticoduodenectomy (PPPD) in 2 (7.1%) and duodenum-preserving pancreatic resection, central pancreatectomy, tumor enucleation, and near-total pancreatectomy in 1 patient each (3.5%).

### 3.4 Surgical outcomes

After surgery, one patient required re-operation because of a postoperative pancreatic fistula (POPF) after enucleation of a tumor in the head of the pancreas. Additionally, another POPF (grade B) occurred in the head, body, and tail of the pancreas in 3, 1, and 1 patients, respectively, requiring the drainage of the collected fluid. The majority of patients exhibited Clavien–Dindo grade II complications. One patient died during the immediate postoperative period owing to intraoperative and postoperative uncontrollable hemorrhage in the pancreatic tail (Figure 1; patient number 4, malignancy pheochromocytoma). According to the tumor location, there was no significant difference in the length of hospital stays (*p* = 0.485). Tumor rupture occurred in four patients with SPNs. Among these, the tumor ruptured preoperatively and was located in the tail of the pancreas in one patient, whereas it ruptured during surgery in three patients; the rupture occurred in the neck in two patients and the tail of the pancreas in one (Table 2). Regarding pathologic diagnosis, the tumor was benign in 25 patients (89.2%) and malignant in 3 (10.7%) (Table 3). The most common benign tumor was SPN, which was noted in 20 (71.4%) patients, followed by a mucinous cyst, nesidioblastosis, pseudocyst, duplication cyst, and benign cyst, each observed in 1 patient (3.5%). The malignant tumors included pancreatoblastoma, solid pseudopapillary carcinoma, and malignant pheochromocytoma, each noted in 1 patient (3.5%).

**Table 3:** Pathologic diagnosis.

Pathologic diagnosis	Number (*N* = 28)
**Benign**	**25** **(89.2%)**
SPN	20 (71.4%)
Mucinous cyst	1 (3.5%)
Nesidioblastosis	1 (3.5%)
Pseudocyst	1 (3.5%)
Benign cyst	1 (3.5%)
Duplication cyst	1 (3.5%)
**Malignant**	**3** (**10.7%)**
Pancreatoblastoma	1 (3.5%)
Solid pseudopapillary carcinoma	1 (3.5%)
Malignant pheochromocytoma	1(3.5%)

SPN, solid pseudopapillary neoplasm.

### 3.5 Long-term outcomes and postoperative growth

Among the 28 patients who underwent pancreatic surgery for pancreatic neoplasms, 1 died in the immediate postoperative period due to intraoperative uncontrollable hemorrhage and 27 remained alive after a median follow-up time of 6.1 years (range: 0.3–15.6 years). During the follow-up period, among patients with SPN, 4 experienced rupture and 2 experienced recurrences. In addition, the patient with solid pseudopapillary carcinoma (Figure 1, patient number #2) had metastatic recurrence, requiring liver transplantation 5 years after pancreatic resection. Adjuvant chemotherapy was administered to 6 patients (21.4%) with pancreatoblastoma (3.5%), solid pseudopapillary carcinoma (3.5%), and SPN (14.2%) after surgery, and no mortality was noted during the follow-up period. The patient's postoperative growth was evaluated using the z-score of weight and height based on the patient's individual growth chart at the time of the last follow-up. Among 28 patients, 4 lacked follow-up data on postoperative growth. When observed according to tumor location, in the pancreatic head, the median weight z-score was 0.94 (range: −0.4–2.7) and the median height z-score was 0.6 (range: −0.3–1.9); in the pancreatic body, the median weight z-score was 0.38 (range: 1.6–2.1) and the median height z-score was −0.1 (range: −1.1–0.8); and in the pancreatic tail, the median weight z-score was 0.09 (range: 1.3–2.7) and the median height z-score was 0.04 (range: 1.1–1.3). No significant impact on growth after pancreatectomy was detected based on tumor location (weight z-score *p*-value = 0.487; height z-score *p*-value = 0.915) (Table 2).

## 4 Discussion

In this study, we analyzed the data of 28 pediatric patients with pancreatic tumors treated over a 15-year period in a single institution and shared our clinical treatment experiences.

We found that pancreatic tumors in pediatric patients are generally asymptomatic and incidentally diagnosed during examinations for other medical conditions. The frequency of symptoms in our study was similar to those of previous studies (8); therefore, it may be pertinent for pediatricians to pay attention to these symptoms when examining patients.

Currently, surgery remains the cornerstone of any curative approach, with the exception of lymphoma (9). A previous study on tumor surveillance in 58 cases of malignancy in pediatric patients reported that surgery is an independent predictor of survival (10). The extent of surgical resection required for complete debulking is dictated by tumor location and histopathologic diagnosis. While radical surgical resection is the gold standard treatment even at the cost of aggressive resections, as it is associated with good prognosis and survival rates (1, 2, 11, 12), considering the histological characteristics of the pancreatic tumor in pediatric patients, blind radical resection may affect the endocrine and exocrine functions of the pancreas. In our study, one tumor of small size in a patient whose enucleation was located at the pancreas head was suggested to be possibly benign on imaging. Therefore, minimal resection was performed with the goal of preserving pancreatic function.

We evaluated data from three patients with malignant tumors (pancreatoblastoma, solid pseudopapillary carcinoma, and malignant pheochromocytoma), all of whom had a rather unfavorable prognosis. The patient with solid pseudopapillary carcinoma underwent adjuvant chemotherapy; however, at the 5-year follow-up, recurrence was observed in the liver resulting in the requirement for liver transplantation. At the most recent follow-up, the patient had remained recurrence-free. Unfortunately, the patient with malignant pheochromocytoma experienced massive bleeding during surgery, and despite efforts to control the bleeding postoperatively, the patient succumbed to mortality. In contrast, we found that pediatric pancreatic neoplasms such as benign and low-grade malignant tumors exhibit diversity. The most common histological subtype was SPN, accounting for 71.4% of all tumors; this incidence was similar to that published in most studies (2, 3, 9). Additionally, we observed other histological subtypes including a mucinous cyst, nesidioblastosis, pseudocyst, duplication cyst, and benign cyst in one patient each. Discussing the risks and benefits of the extension of surgical resection is particularly difficult owing to a lack of published data. We observed that different extents of surgical resections were possible even for tumors in similar locations. Considering the histological characteristics of pancreatic tumors, it may be necessary to consider postoperative favorable prognosis and determine the appropriate surgical method and extent of resection. Previous studies have reported an overall morbidity of 20%–50% and a POPF rate of 0.0%–25% in adults (13–15), whereas recent reports from the US and Italy have reported high rates of postoperative complication in pediatric pancreaticoduodenectomy (PD). Nevertheless, radical resection methods such as PD have been reported to be effective in treating lesions located in the pancreatic head (1, 16). Owing to the rarity of primary pancreatic head tumors, the management of pancreatic head tumors is challenging for pediatric surgeons who lack experience. In our study, one patient with pancreatoblastoma was diagnosed at an advanced stage; hence, neoadjuvant chemotherapy was performed. The patient remained disease-free at follow-up after successful chemotherapy and successful PPPD with complete tumor resection.

Previous studies have reported short-term complication rates of 23%–51% (3, 17). In our study, one patient required re-operation because of a POPF after enucleation of the pancreatic head mass and three patients required fluid drainage for POPF grade B. However, no severe (Clavien–Dindo grade III) complications were observed. Compared with previous studies, our study demonstrated a low rate of postoperative complications, which may be attributed to the diverse extents of surgical resection.

Contrary to our expectations, when height and weight were confirmed for each extension of resection (head, body, and tail) in patients who could be followed up, none of them had severe growth problems. This result is similar to previously reported findings; Chiara et al. (3) first reported the postoperative growth of pediatric patients after pancreatic surgery. They found that the median height and weight development in their cohort was slightly decreased (−0.5 SD). However, none of our patients developed a significant growth deficit as observed at the last follow-up. Endocrine and exocrine function are also important outcomes after pancreatectomy. In adults, the reported rates for exocrine insufficiency are 56%–73%, and the rates of endocrine insufficiency following PD are 20%–25% (7). In their study, Cheng et al. (3) reported on long-term follow-up outcomes of 14 pediatric patients who underwent pancreatectomy, revealing that two patients (14.2%) experienced postoperative fat malabsorption. However, no evidence of endocrine failure was noted, which confirms the benefits of preserving as much normal pancreatic tissue as possible.

One limitation of our study was its single-center retrospective design. There was also an absence of data on endocrine and exocrine function for long-term follow-up patients that limited the evaluation of long-term postoperative pancreatic function. Additionally, there were no data on the surgical outcomes for each histological specific. Future multi-center studies with a large sample size are required to further elucidate the characteristics and prognosis of pancreatic neoplasms in children.

## 5 Conclusions

Over the past 15 years, we have treated various types of pancreatic neoplasms in children. In this case series, benign histological diagnosis was dominant and various extents of surgical resection were performed. The surgical treatment for pediatric pancreatic neoplasms appears to be safe and effective. However, considering the long-term prognosis of these patients, it is important to determine the appropriate extent of surgical resection based on the location of the tumor. Despite various histological diagnoses, there is a lack of research on the specific histological characteristics and prognostic factors associated with pancreatic tumors. Therefore, future studies on the histological features of pediatric tumors and their association with outcomes are necessary.

## Data Availability

The original contributions presented in the study are included in the article/Supplementary Material, further inquiries can be directed to the corresponding author.
